# Immunocyte Infiltration Analysis and Immunohistochemistry Identify EVL as a Potential Prognostic Biomarker for Pancreatic Cancer

**DOI:** 10.3390/jpm13030433

**Published:** 2023-02-28

**Authors:** Yan Du, Lin Zhu, Xin Li, Huaqing Shi, Wenkai Jiang, Wence Zhou

**Affiliations:** 1 The Second School of Clinical Medicine, Lanzhou University, Lanzhou 730030, China; 2 The First Clinical Medical College, Lanzhou University, Lanzhou 730000, China; 3 Department of General Surgery, Lanzhou University Second Hospital, Lanzhou 730030, China

**Keywords:** pancreatic cancer, EVL, prognosis, immune infiltration, biomarker

## Abstract

Ena-VASP-like (EVL), a member of the Enabled/vasodilator stimulated phosphoprotein family, is functionally expressed in various cancers. This study explored the prognostic value and potential mechanism of EVL in pancreatic cancer (PC). RNA-seq obtained from The Cancer Genome Atlas (TCGA) and Gene Expression Omnibus (GEO) databases were used to evaluate EVL expression differences, and clinical samples were collected for validation. The prognostic value of EVL was evaluated by survival data obtained from TCGA and clinical samples. The biological pathways involved in EVL were evaluated by functional enrichment analysis such as GO, KEGG, and GSEA. We used immune infiltration analysis to estimate the correlation between EVL and tumor-infiltrating immune cells (TICs). The expression of EVL is down-regulated in PC tissues, which is an independent factor affecting survival time. Survival analysis suggested EVL-high expression was associated with good prognosis in PC patients. The results of the enrichment analysis suggested that the biological function of EVL was closely related to the immune mechanism. Tumor immune infiltration analysis showed that high expression of EVL was accompanied by high levels of immune infiltration. Furthermore, EVL was strongly correlated with the content of immune cells such as CD8+ T cells, B cells, regulatory T cells, CD4+ Tem cells, and follicular Th cells. EVL is a potential independent prognostic marker and immunotherapy target for PC. Mechanistically, EVL may affect the prognosis by extensively promoting immune cell infiltration, including strengthening the anti-tumor immune response of CD8+ T cells.

## 1. Introduction

Pancreatic cancer (PC) is a highly lethal malignant tumor with an incidence of approximately 0.4% per year due to the growth of the global population and the spread of public health epidemics [1]. The 5-year overall survival rate of PC is about 10%, which has improved in recent years. However, it remains the worst prognosis among common malignant solid tumors [2]. Due to the lack of early clinical symptoms, PC patients are usually in the advanced stage when they are diagnosed, and more than 80% of PC patients are not suitable for surgical resection [3]. Unresectable PC patients require chemotherapy, radiotherapy, and immune checkpoint blockade therapy. However, tumor drug resistance, connective tissue hyperplasia, and immunosuppressive microenvironment increase the difficulty of systemic treatment [4]. Because of the rapid progression of the tumor, few patients achieved complete pathological remission [5]. Therefore, it is significant to look for diagnostic markers and predict new therapeutic targets for PC patients to improve their outcomes.

Enabled/vasodilator stimulated phosphoprotein (Ena/VASP) proteins are a family of proteins involved in the regulation of cell actin, including protein-enabled homolog (Mena), VASP, and Ena-VASP-like (EVL) [6]. The protein family can affect the length, aggregation, and network branching of actin filaments, and they are also related to the remodeling of the cytoskeleton and cell movement [7]. EVL has received more attention in recent studies. A study focusing on endothelial cells showed that EVL can regulate the production of vascular buds through the VEGF signaling pathway [8]. In another study, EVL was involved in antigen stimulation responses and target cell adhesion in the cytotoxic killing effects mediated by Natural killer (NK) cells [9]. In addition, EVL was closely related to the pathogenesis of breast cancer. Mouneimne et al. indicated that EVL-mediated actin polymerization can inhibit the cell movement of breast cancer, thereby reducing the invasion ability of tumors [10]. Tavares et al. found that the up-regulated EVL expression in early breast cancer could promote cell sclerosis and cell proliferation, but the expression level of EVL was reversed and down-regulated during tumor progression to promote tumor cell migration [11]. In our previous study, we performed expression profiling on plasma samples of PC patients and found that EVL expression was suppressed in peripheral blood and was associated with a better prognosis of patients [12]. In this study, we will further evaluate the expression level and prognostic significance of EVL in tissue samples from PC patients, and explore the potential mechanisms of EVL in PC.

## 2. Materials and Methods

### 2.1. RNA-Seq Data Download

The Cancer Genome Atlas (TCGA) database contains gene expression data for 178 PC tissues and 4 normal pancreatic tissues [13]. The University of California Santa Cruz (UCSC) database collates and standardizes the data in TCGA, which can be directly obtained for differential expression analysis of genes [14]. We logged into the UCSC database on 13 October 2021, to obtain the TCGA Pan-Cancer dataset, all in HTseq-FPKM format. We screened the sequencing data in the Gene Expression Omnibus (GEO) to further verify the results of gene expression analysis. The inclusion criteria were: PC tissue samples (*n* > 30), normal pancreatic tissue samples (*n* > 15), and platform file gene ID that could be converted into commonly used gene names [15].

### 2.2. Clinical Data Download

The clinical information of PC patients was obtained from TCGA. After deleting samples with missing survival data, a total of 178 patients were included. We extracted the expression level of EVL in different clinical groups: histologic grade (G1 vs. G2 vs. G3/4), pathologic stage (stage1 vs. stage2 vs. stage3/4), T stage (T1/2 vs. T3/4), N stage (N0 vs. N1), and M stage (M0 vs. M1). The Wilcox test was used to compare the outcomes between groups. Univariate and multivariate Cox analyses were used to evaluate the prognostic value of EVL and clinical characteristics for PC patients. The Kaplan-Meier survival curve was drawn to evaluate the difference in the survival status of patients between the EVL-high and EVL-low groups. Based on the results of Cox regression analysis, a nomogram model was established to predict overall survival in PC.

### 2.3. Functional Analysis of Co-Expressed Genes

In the PC data set of LinkedOmics, the co-expressed genes of EVL were obtained through correlation analysis [16]. The absolute value of the correlation coefficient ≥ 0.5 and *p* < 0.05 were defined as meaningfully co-expressed genes. Gene Ontology (GO) analysis and Kyoto Encyclopedia of Genes and Genomes (KEGG) can systematically analyze the cell biochemical pathways involved in the genome. We used ClusterProfiler R package to perform GO and KEGG enrichment analysis of EVL co-expressed genes.

### 2.4. Functional Analysis of EVL-Related DEGs

The PC samples were divided into EVL-high and EVL-low groups according to the median value of EVL expression levels. Differentially expressed genes (DEGs) were obtained by the Wilcox test between EVL-high and EVL-low groups. The screening threshold was defined as the *p*-value < 0.05 and the absolute value of Log Fold Change (LogFC) > 0.6. To further annotate the function of EVL in PC, we performed GO and KEGG enrichment analyses for EVL-related DEGs.

### 2.5. Gene Set Enrichment Analysis (GSEA) of EVL

The gene expression matrix of TCGA-PC was converted to the standard input file format of GSEA, and the functional enrichment analysis between EVL-high and EVL-low groups was performed through the GSEA software (4.0.2) [17]. The c2.cp.kegg.v7.4.symbols.gmt data set and the c5.go.v7.4.symbols.gmt data sets were used as controls, and the number of permutations was set to 1000. Significantly enriched pathways need to meet the following criteria: false discovery rates (FDR) < 0.25, the absolute value of the normalized enrichment score (NES) > 1, and the nominal *p*-value (NOM *p*-value) < 0.05.

### 2.6. Correlation Analysis of Immune Infiltration

We analyzed the relationship between EVL expression and PC immune cell infiltration by the ssGSEA algorithm [18]. Specifically, the GSVA R package was used to obtain the content of 28 TICs in each PC sample. Then, the infiltration levels of 28 TICs were compared between EVL-high and EVL-low groups. Finally, Pearson correlation analysis was used to evaluate the correlation between EVL expression levels and the content of 28 TICs. Additionally, we also assessed the correlation between EVL expression levels and immune-related gene sets, including MHC genes, immune activating genes, chemokine receptors, and chemokines.

### 2.7. Patients and Cell Lines

In total, 85 PC tissues and 50 adjacent normal tissues were used for further analysis. 5 PC tissues and 5 normal tissues were used for qRT-PCR. 80 PC tissues and 45 normal tissues were used for immunohistochemistry. All samples had been diagnosed with pancreatic ductal adenocarcinoma via postoperative pathology and did not receive preoperative chemotherapy or radiotherapy. Patients provided written informed consent to participate in this study, and the Ethics Committee of the First Hospital of Lanzhou University approved all protocols involving human samples discussed herein. PC cell lines (ASPC-1, SW1990, BXPC3 and PANC-1) and human pancreatic cell lines (HPNE) were obtained from the Shanghai Institute of Nutrition and Health (Shanghai, China).

### 2.8. RNA Extraction and qRT-PCR

RNA was isolated from samples using TRIzol, after which the PrimeScript RT Reagent Kit was employed to prepare cDNA. A StepOne Real-Time PCR Instrument (Ap-plied Biosystems, Nyack, NY, USA) was used for all qRT-PCR analyses. Relative gene expression was analyzed using the 2^^-ΔΔCt^ method, with GAPDH being employed for normalization purposes. Primers used in this study were forward: CTTCCGTGATGGTCTACGATG and reverse: TGCAACTTGACTCCAACGACT, and analyses were repeated three times.

### 2.9. Western Blotting

RIPA buffer (Beyotime, Shanghai, China) supplemented with protease inhibitors (Roche, Pleasanton, CA, USA) was utilized to lyse cells. Equal amounts of protein extracts were then separated via 10% SDS-PAGE (Beyotime) and transferred onto 0.4 μm PVDF membranes (Roche) that were locked with 5% non-fat milk and probed overnight with anti-EVL (Proteintech, Wuhan, China) at 4 °C. Blots were then probed with secondary goat anti-rabbit IgG for 2 h at room temperature, after which protein bands were detected with an ECL reagent. GAPDH was utilized as a loading control for these analyses.

### 2.10. Immunohistochemical Assay

EVL antibody (Proteintech 1:500) was used for immunohistochemistry. Semi-quantitative evaluation of immunohistochemical results was performed through observation by two experienced pathologists. The percentage of positive cells (EXT) was divided into 4 grades: 0 (0%), 1 (<10%), 2 (10–50%), and 3 (>50%). There were four grades of the staining intensity (INT): 0 (no staining), 1 (yellow), 2 (tan), and 3 (brown). The relative expression index was calculated by the following formula: (EXT * INT). Scores < 4 were low expressions, and scores ≥ 4 were high expressions.

### 2.11. Statistical Analysis

Software used for statistical analysis of data: R (version 4.0.2, R Foundation, Vienna, Austria), SPSS (version 25.0, IBM SPSS Inc., New York, NY, USA), Prism (version 8.0.2). Tools used for data visualization: ggplot2 R package, Prism (version 8.0.2, GraphPad Inc., California, CA, USA). The chi-square test was used to analyze the differences in clinical characteristics between EVL high and low expression groups. T-test (normal distribution and homogeneity of variance) and Wilcoxon rank-sum (abnormal distribution or non-homogeneity of variance, RNA-seq data) test were used to compare the differences in EVL expression levels between different groups. The Spearman test was used to evaluate the correlation between EVL expression and clinical characteristics. The Kaplan-Meier survival curve, Univariate and multivariate Cox analyses were used to assess the prognostic value of EVL in clinical samples again. The cox.zph function in the SURVIVAL R package was used to verify the proportional hazards (PH) assumption for each variable introduced into the Cox model, and the variables were considered to satisfy the PH assumptions when *p* > 0.05. *p* < 0.05 was considered statistically significant.

## 3. Results

### 3.1. EVL Was Down-Regulated in PC Tissues

**Figure 1:**
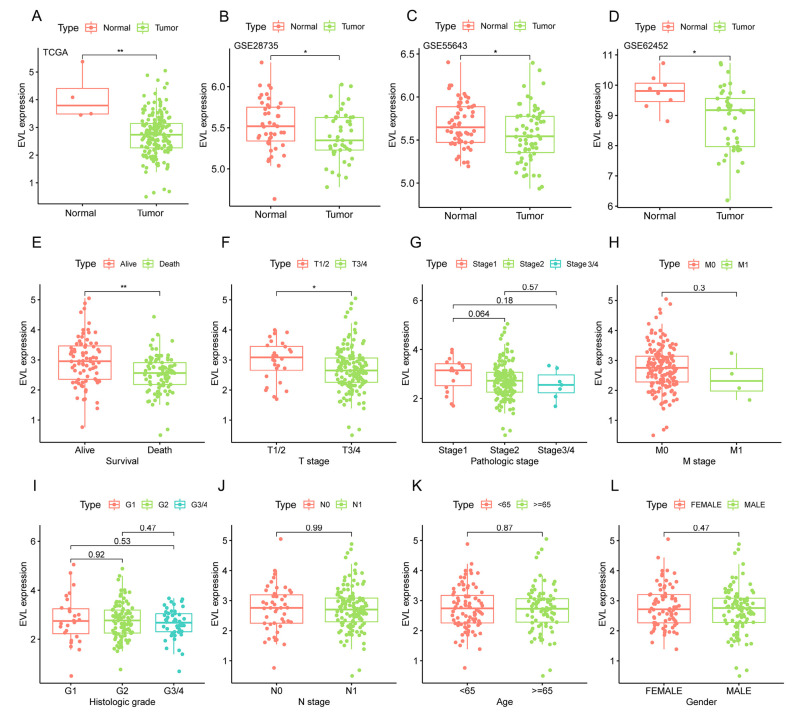
Comparison of EVL expression between different groups in pancreatic cancer. EVL, Ena-VASP-like. * *p* < 0.05, ** *p* < 0.01.

**Figure 2:**
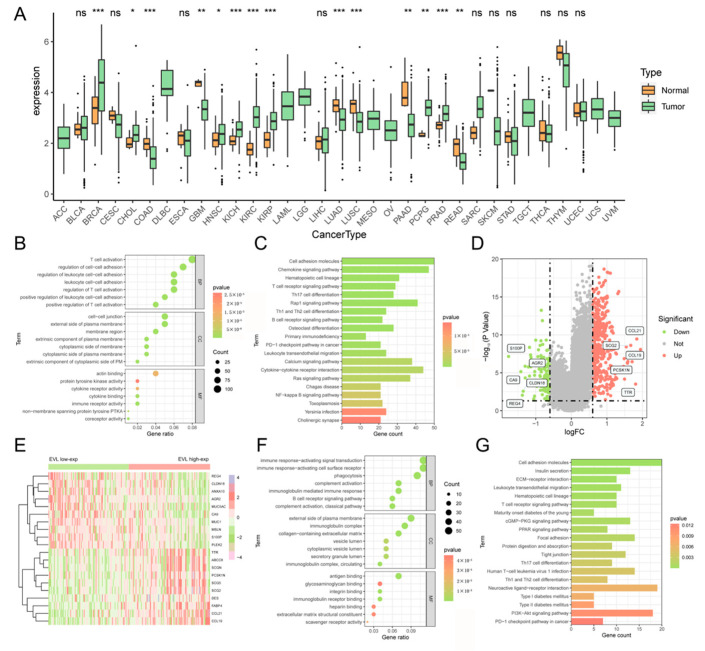
Comprehensive enrichment analysis associated with EVL. (**A**) EVL pan-cancer analysis in 33 types of tumors. (**B**,**C**) GO and KEGG analysis based on EVL co-expressed genes. (**D**) DEGs between EVL-high and EVL-low groups. (**E**) The heatmap of the top 20 DEGs. (**F**,**G**) GO and KEGG analysis based on EVL-related DEGs. EVL, Ena-VASP-like; GO, gene ontology; KEGG, Kyoto encyclopedia of genes and genomes; DEGs, differentially expressed genes. * *p* < 0.05, ** *p* < 0.01, *** *p* < 0.001, ns, not significant.

The results of the differential expression analysis based on TCGA showed that the expression level of EVL in the tumor group was lower than that in the normal group (*p* < 0.01, Figure 1A). To further verify this result, we extracted the sequencing expression data of EVL in the GEO database. The analysis results of GSE28735, GSE55643, and GSE62452 showed that the expression level of EVL in PC tissues showed a downward trend (all *p* < 0.05, Figure 1B–D). In addition, we also evaluated the EVL expression between different groups based on clinical characteristics. The results showed that the EVL expression levels in the T1/2 group and the alive group were higher than those in the T3/4 group and the death group (all *p* < 0.05, Figure 1E,F). The expression of EVL was not significantly different among the groups of histologic grade, pathologic stage, N stage, M stage, age and gender (Figure 1G,H). In addition, the results of the pan-cancer analysis showed that 8 of the 33 types of tumors had higher EVL expression levels than the corresponding normal tissues, and 6 types of tumors had lower EVL expression levels than the corresponding normal tissues (Figure 2A).

### 3.2. The Function of EVL Co-Expressed Genes Related to Immune Pathways

We obtained the genes set associated with EVL expression in the PC through the LinkedOmics database and screened out 1555 strong correlation genes with statistical significance for functional enrichment analysis. The results of GO analysis show that the most significant terms were closely related to immune cell functions, including T cell activation, leukocyte cell-cell adhesion, lymphocyte proliferation, and regulation of leukocyte proliferation (Figure 2B). The results of the KEGG enrichment analysis also contained a large number of immune-related pathways, including immune cell pathways (T cell receptor signaling pathway, Th17 cell differentiation, B cell receptor signaling pathway), and pathways that regulated the immune system (Hematopoietic cell lineage, Primary immunodeficiency, Osteoclast differentiation). In addition, some cellular pathways (NF-kappa B signaling pathway, Ras signaling pathway) that affect tumor occurrence and development also appeared in the enrichment results (Figure 2C). 

### 3.3. The Function of EVL-Related DEGs Related to Immune Pathways

DEGs between EVL-high and EVL-low groups were also used for functional enrichment analysis. We obtained 120 down-regulated and 443 up-regulated DEGs (Figure 2D). The expression levels of TOP10 up-DEGs and TOP10 down-DEGs are displayed in the heatmap (Figure 2E). GO and KEGG enrichment analyses indicated that DEGs involved biological effects and signaling pathways were closely related to the immune system (TOP 20 significant GO term and KEGG pathways were respectively shown in Figure 2F,G). Immune-related enrichment results included immunoglobulin mediated immune response, immunoglobulin receptor binding, B cell receptor signaling pathway, leukocyte transendothelial migration, T cell receptor signaling pathway, Th1 and Th2 cell differentiation, and PD-1 checkpoint pathway in cancer.

### 3.4. EVL Positively Regulates the Immune Pathway

**Figure 3:**
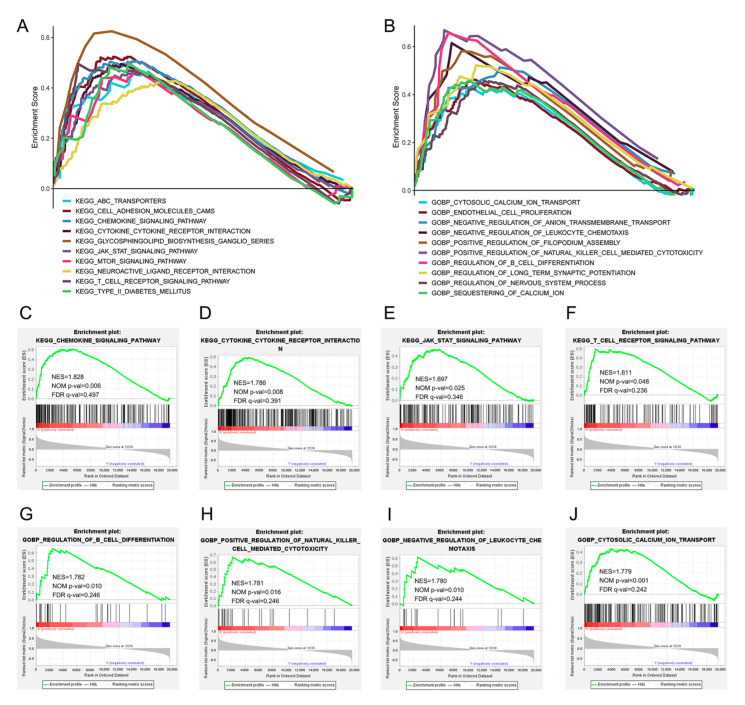
GSEA based on the EVL-high and EVL-low groups. (**A**) Top 10 upregulated GSEA-GO terms. (**B**) Top 10 upregulated GSEA-KEGG pathways. (**C**–**J**) Immune system-relevant signaling pathways and biological processes in GSEA results. EVL, Ena-VASP-like; GSEA, gene set enrichment analysis; NES, normalized enrichment score; NOM *p*-val, nominal *p*-value; FDR q-val, false discovery rate q-value; GO, gene ontology; KEGG, Kyoto encyclopedia of genes and genomes.

The results of enrichment analysis of related genes suggested that the function of EVL in PC may be potentially related to the immune system. Therefore, we further assessed whether EVL was involved in the regulation of immune pathways through GSEA. The PC samples were divided into high-expression and low-expression groups according to the expression level of EVL for enrichment analysis. The ten pathways with the largest NES value in the analysis results of GSEA-KEGG and GSEA-GO were shown in Figure 3A,B, respectively. These results suggested that when EVL was highly expressed, the activity of a variety of immune-related biological processes increases, including the Regulation of B cell differentiation, Regulation of natural killer cell mediated cytotoxicity, and Negative regulation of leukocyte chemotaxis (Figure 3H,I). EVL can also positively regulate the activity of cell surface receptors and cytokines, including the Chemokine signaling pathway, Cytokine cytokine receptor interaction, T cell receptor signaling pathway, and Cytosolic calcium ion transport (Figure 3C,D,F,J). In addition, EVL can activate the JAK-STAT signaling pathway associated with tumors (Figure 3E). These results further verify that EVL may exert an anti-tumor effect by regulating immune cell function and promoting immune signal transduction.

### 3.5. EVL Promotes the Immunocyte Infiltration of PC

**Figure 4:**
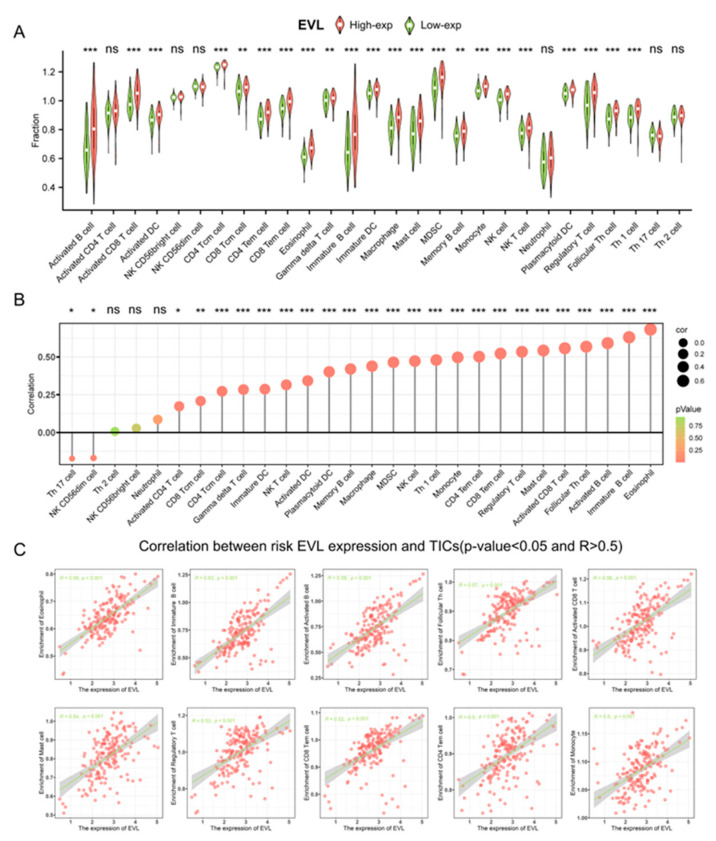
EVL expression is associated with immune cells infiltration in pancreatic cancer. (**A**) Comparison of the different TICs infiltration levels under high and low EVL expression conditions. (**B**) Correlation analysis between the expression of EVL and the content of TICs. (**C**) 10 TICs showed strong correlation with EVL expression. EVL, Ena-VASP-like; TICs, tumor-infiltrating immune cells. * *p* < 0.05; ** *p* < 0.01; *** *p* < 0.001; ns, not significant.

**Figure 5:**
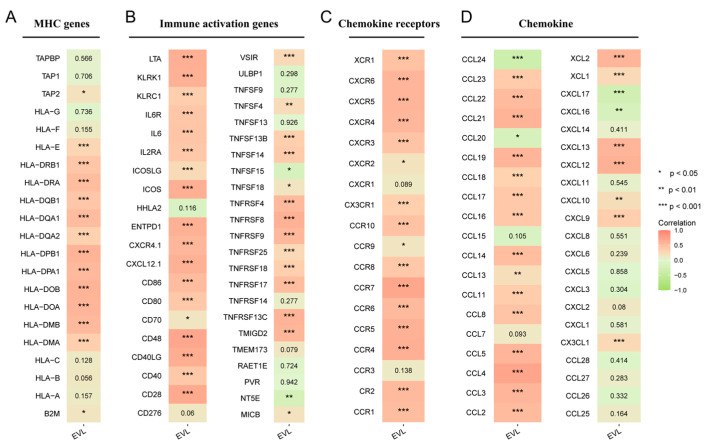
Correlation of EVL with immune-related gene sets. Correlation of EVL and MHC genes (**A**), immune activation genes (**B**), chemokine receptors (**C**) and chemokines (**D**). EVL, Ena-VASP-like. * *p* < 0.05; ** *p* < 0.01; *** *p* < 0.001.

We analyzed the relationship between EVL expression and immunocyte infiltration in PC tissue samples. The contents of various immune cells in patients were obtained through GSVA R packs, and it was found that the content of 22 TICs in the EVL high expression group was significantly higher than the EVL low expression group Group (all *p* < 0.01, Figure 4A). The results of the Pearson correlation analysis showed that the content of 23 types of TICs was positively correlated with the expression level of EVL, and the content of 2 TICs was negatively correlated with the EVL expression (all *p* < 0.05, Figure 4B). Among them, 10 TICs showed a strong correlation with EVL expression, including monocyte, effector memory CD4+ T cell (CD4+ Tem cell), effector memory CD8+ T cell (CD8+ Tem cell), regulatory T cell, mast cell, activated CD8+ T cell, follicular Th cell, activated B cell, immature B cell, eosinophil (all R > 0.5 and *p* < 0.05, Figure 4C). We further explored the co-expression relationship between EVL and immune-related gene sets. The results showed that MHC genes and immune activation genes showed a positive co-expression relationship with EVL expression (Figure 5A,B). In addition, most chemokine receptors and chemokines were positively correlated with EVL expression (Figure 5C,D). These outcomes proved the results of the previous step of functional analysis and suggested that EVL could promote the recruitment of immune cells in the PC microenvironment and improve the patient’s immunocyte infiltration level.

### 3.6. EVL Indicates a Good Prognosis for PC Patient

**Figure 6:**
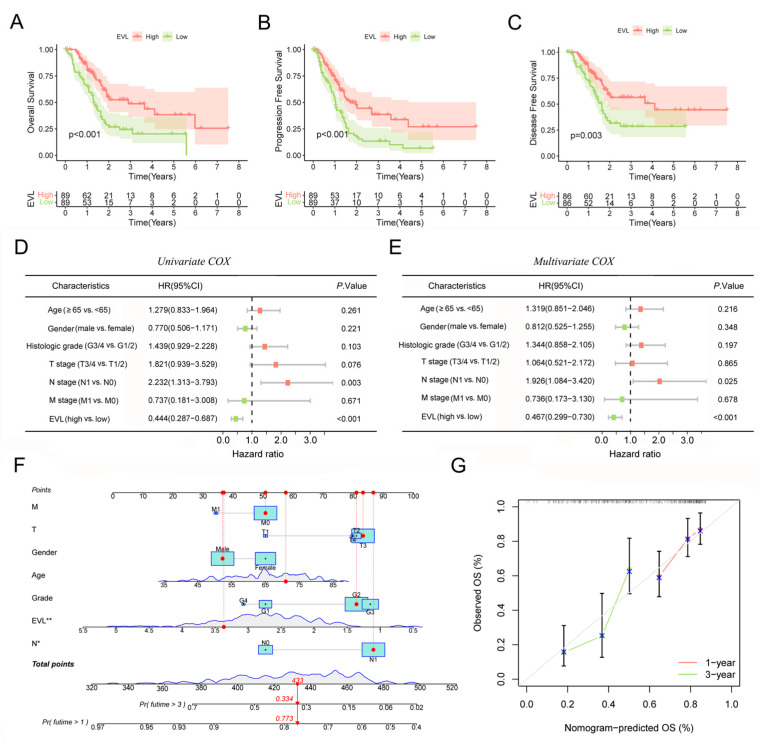
EVL indicates a good prognosis for pancreatic cancer patients. (**A**–**C**) Kaplan–Meier survival curves of overall survival time, progression-free survival time and disease-specific survival time; (**D**,**E**) Univariate and multivariate Cox analysis of EVL and clinical characteristics. (**F**,**G**) The nomogram model and calibration plot of EVL and clinical characteristics. EVL, Ena-VASP-like. * *p* < 0.05; ** *p* < 0.01.

The previous results showed that EVL expression was inhibited in tissue samples of PC patients. We further analyzed the impact of EVL on the prognosis of patients. The results of the Kaplan-Meier survival curve showed that the high expression level of EVL was associated with a longer overall survival time, a longer progression-free survival time and a longer disease-specific survival time (all *p* < 0.01, Figure 6A–C), which revealed that EVL-high expression predicted a good prognosis for PC patients. All variables included in the COX regression analysis are consistent with the PH assumptions. The results of the COX univariate analysis showed that N staging and EVL were factors that affected patient survival (Figure 6D), while COX multivariate results showed that N staging and EVL level were independent prognostic factors for patient survival (Figure 6E). We further assessed the prognostic value of EVL by constructing a nomogram. The C-index of the nomogram model was 0.665 (95% CI: 0.602–0.728), indicating that the model had some predictive power for prognosis (Figure 6F). The calibration plot showed that the 1-year and 3-year bias correction lines were close to the 45° diagonal line, indicating that the predicted survival time was basically consistent with the theoretical value (Figure 6G). The results of this part suggest that EVL has great potential in the prognostic evaluation of PC patients.

### 3.7. Validating the Expression Differences of EVL

**Figure 7:**
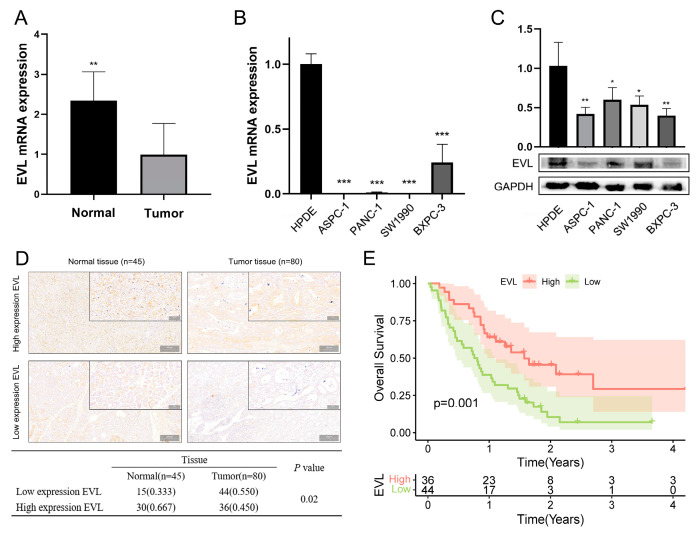
Validating the expression differences of EVL. (**A**,**B**) qRT-PCR analysis of EVL expression in clinical samples and PC cell lines. (**C**) WB analysis of EVL expression in PC cell lines. (**D**) EVL expression was determined by immunochemistry. (**E**) Kaplan-Meier survival curve between the EVL-high and EVL-low groups. * *p* < 0.05; ** *p* < 0.01; *** *p* < 0.001.

We collected 5 PC tissues and 5 adjacent normal tissues to verify the differential expression of EVL in clinical samples. The results of qRT-PCR showed that the expression of EVL in normal tissues was higher than that in PC tissues (Figure 7A). The results showed that both mRNA and protein expression levels of EVL were up-regulated in tumor cell lines (Figure 7B,C) after evaluating the expression of EVL in four PC tumor cell lines (ASPC1, SW1990, BXPC3, PANC1) and one normal cell line (HPDE). In addition, we detected EVL expression in 80 PC tissues and 45 normal tissues by immunohistochemistry. The results showed that the expression level of EVL was down-regulated in PC tissues (44 cases with low expression, 55%), while the expression of EVL was up-regulated in normal tissues (30 cases with high expression, 66.7%). The expression of EVL was abnormally down-regulated in PC tissues compared with normal tissues (Figure 7D).

### 3.8. Validating the Prognostic Value of EVL

The clinical characteristics of 80 PC tissues are shown in Table 1. The results show that the tumor histologic grade in the EVL-high and EVL-low groups are concentrated in the G1 and G2/3 stages, respectively (*p* < 0.05). There were no statistical differences in other characteristics between the EVL-high and EVL-low groups (Table 1). The results of Spearman analysis suggested that EVL expression level was negatively correlated with histologic grade (Table 2). Univariate and multivariate Cox analysis suggested that EVL, age and N stage were independent factors affecting the survival time of PC patients, and EVL was a protective factor (Table 3). The variables included in the Cox model are all consistent with the PH hypothesis. Consistent with TCGA data, the Kaplan-Meier survival curve of clinical samples showed that the survival time of PC patients in the EVL-high group was longer than that in the EVL-low group (Figure 7E).

## 4. Discussion

EVL is a member of the Ena/VASP family, and recent studies showed that it was related to the biological processes of a variety of tumors. In gastric signet-ring cell carcinoma, the deletion of the Estrogen Receptor beta gene promotes tumor invasiveness through the mTOR-Arpc1b/EVL signaling pathway, resulting in a higher T stage [19]. The expression level of EVL in cervical cancer was lower than the matched normal tissue, and also lower than the expression level of cervical intraepithelial neoplasia [20]. Our previous research results showed that the expression of EVL was inhibited in the plasma samples of PC patients, and the overall survival time of patients in the high EVL expression group was better than patients in the EVL low expression group [12]. In the current study, we found that EVL expression was abnormally downregulated in PC tumors through the TCGA-PC dataset, which was validated in GEO microarray data and clinical samples. EVL also showed low expression in PC cell lines compared to normal pancreatic cell lines. EVL has sufficient potential for prognostic assessment in PC patients. First, the OS time, PFS time, and DSS time of patients in the EVL high expression group were better than those in the EVL low expression group. Multivariate COX analysis showed that EVL was an independent prognostic factor for patients. Then, the nomogram constructed based on EVL also reflects the prognostic predictive ability of EVL. Enrichment analysis showed that EVL was involved in a variety of tumor-related pathways and immune system-related functional processes. These results suggest that EVL, as a tumor suppressor gene in PC, may be involved in antitumor immune mechanisms.

The reduced infiltration and activity inhibition of immune effector cells leads to the low immunogenicity of the PC, which has a negative effect on the treatment effect and prognosis of patients [21]. In addition to surgery in eligible patients, gemcitabine and FOLFIRINOX are currently common chemotherapy regimens used to prevent PC progression. However, the application of these chemotherapy strategies is limited due to severe side effects and individual differences [22]. With the research progress of tumor microenvironment, immunotherapy against solid tumors has become a hotspot in tumor treatment. In the present study, we comprehensively assessed the regulatory functions of EVL in complex antitumor immunity based on functional enrichment analysis, immune cells infiltration analysis and immune genes co-expression analysis. EVL is involved in various immune biological processes and immune pathways in PC. The expression of most immune-related genes is synergistic with EVL, including MHC genes, chemokine receptors and chemokines. Crucially, EVL displayed a strong association with TICs, including key regulators of antitumor immunity (CD8+ T cell, B cell, regulatory T cell, CD4+ Tem cell, follicular Th cell).

CD8+ cytotoxic T lymphocytes are the main subgroup of CD8+ T cells, which can kill tumor cells by secreting effectors such as perforin, granzymes, and TNF [23]. The CD8+ memory T cell subsets can mediate a continuous anti-tumor immune response and become a barrier to inhibit tumor progression and recurrence [24]. However, the excessive consumption and dysfunction of CD8+ T cells caused by PC tissue weakened these anti-tumor effects [25]. Previous studies showed that EVL played a key role in the migration and transport of T cells [26], so we speculated that the impaired function of CD8+ T cells in the PC may be affected by the inhibition of EVL expression. Previous studies have shown that B cells are involved in regulating the development and progression of solid tumors. In breast and ovarian cancer, B cells are a major component of infiltrating immune cells in the tumor microenvironment, predicting improved patient survival [27]. However, B cell infiltration promotes tumor progression in mouse models, such as EL4 thymoma and MC38 colon cancer [28]. A study showed that EVL had a regulatory effect on the differentiation process of bone marrow hematopoietic cells, induced the production of lymphocytes, and had up-regulated expression in both B cell and T cell lineages [29]. Our research showed that EVL was involved in the regulation of B cell differentiation and was positively correlated with the content of activated B cells and mature B cells, which suggested that EVL may be related to the development of lymphocytes. 

In addition, the content of follicular Th cells, regulatory T cells and CD4+ Tem cell were also strongly positively correlated with EVL expression. Follicular Th cells are members of the Th subset family and limited studies have involved their function in the PC tumor microenvironment. Follicular Th cells are mainly involved in the interactions between immune cells in lymphoid organs, such as the production of T cell-dependent antibodies and memory B cells [30]. Interestingly, recent studies point to the value of follicular Th cells in tumor prognosis assessment. In both subtypes of breast cancer (HER2+ and ER+/HER2−), high invasive levels of follicular Th cells have good prognostic significance [31]. In non-small cell lung cancer, circulating follicular Th cells are abnormally upregulated and predict poor prognosis [32]. Regulatory T cells played multiple roles in PC. They can promote tumor progression by inhibiting the activity of T cells, while they can suppress tumors by promoting the function of myofibroblastic cancer-associated fibroblasts [33,34]. The Memory CD4+ T cell was a subgroup of immune memory cells with a relatively long survival time and it can mediate the secondary immune response of tumors [35]. The percentage of Memory CD4+ T cells in the unaffected lymph nodes of breast cancer patients was increased, which inhibited tumor progression by hindering lymph node metastasis [36]. Memory CD4+ T cells are a subgroup of immune memory cells that primarily mediate secondary immune responses against tumors [35]. In the non-invaded lymph nodes of breast cancer, the memory CD4+ T cells inhibits tumor progression by hindering lymph node metastasis [36].

The results of the immune-related analysis suggest that the highly expressed EVL promotes the immune infiltration of PC by affecting the content and function of a variety of immune cells. The concept of immune-inflamed tumors was recently proposed to describe tumors with an immune microenvironment infiltrated by high levels of CD4+ T cells and CD8+ T cells. Due to immune-inflamed tumors being highly immunogenic and immunoreactive, tumor patients can benefit from immunotherapy [37]. For cold tumors like PC, it was a good treatment strategy to increase the level of CD8+ T cell infiltration by using local immune activators [38]. Therefore, the close connection between EVL and CD8+ T cells may be a potential target for PC immunotherapy.

## 5. Conclusions

In conclusion, our research showed that EVL can improve the level of immune infiltration of PC, especially the immune function of CD8+ T cells. Downregulation of EVL expression in PC tissues reduced the effect of this anti-tumor immunity, leading to a poor prognosis for patients. Therefore, EVL is a potential prognostic marker for PC patients and a potential target for immunotherapy.

## Figures and Tables

**Table 1:** Clinical characteristics of 80 PC patients.

Characteristics	EVL Expression		*p* Value
	Low, No. of Cases	High, No. of Cases	
Gender			0.503
Female	30 (0.682)	27 (0.75)	
Male	14 (0.318)	9 (0.25)	
Age			0.886
≤65	30 (0.682)	24 (0.667)	
>65	14 (0.318)	12 (0.333)	
CA199			0.747
≤35	12 (0.273)	11 (0.306)	
>35	32 (0.727)	25 (0.694)	
CEA			0.848
≤5.2	35 (0.795)	28 (0.778)	
>5.2	9 (0.205)	8 (0.222)	
T stage			0.154
T1/2	25 (0.568)	26 (0.722)	
T3/4	19 (0.432)	10 (0.278)	
N stage			0.398
N0	24 (0.545)	23 (0.639)	
N1	20 (0.455)	13 (0.361)	
M stage			0.267
M0	42 (0.955)	32 (0.889)	
M1	2 (0.045)	4 (0.111)	
Pathologic stage			0.116
Stage1/2	38 (0.864)	26 (0.722)	
Stage3/4	6 (0.136)	10 (0.278)	
Histologic grade			0.011
G1	9 (0.205)	17 (0.472)	
G2/3	35 (0.795)	19 (0.528)	

**Table 2:** Spearman analysis between EVL and clinical characteristics.

Characteristics	Spearman Correlation	*p* Value
Gender	−0.075	0.509
Age	0.016	0.887
CA199	−0.036	0.751
CEA	0.021	0.85
T stage	−0.159	0.158
N stage	−0.094	0.405
M stage	0.124	0.273
Pathologic stage	0.176	0.119
Histologic grade	−0.284	0.011

**Table 3:** Univariate and multivariate Cox regression analysis.

Characteristics	Univariate Analysis		Multivariate Analysis	
	Hazard Ratio (95% CI)	*p* Value	Hazard Ratio (95% CI)	*p* Value
Gender (male vs. female)	0.984 (0.560–1.731)	0.956	0.867 (0.47–1.6)	0.649
Age (≥65 vs. <65)	1.457 (0.845–2.512)	0.176	1.912 (1.055–3.467)	0.033
T stage (T3/4 vs. T1/2)	2.199 (1.299–3.725)	0.003	1.692 (0.967–2.962)	0.065
N stage (N1 vs. N0)	1.85 (1.099–3.115)	0.021	1.948 (1.103–3.44)	0.022
M stage (M1 vs. M0)	1.528 (0.608–3.840)	0.368	1.89 (0.722–4.946)	0.195
Histologic grade (G3/4 vs. G1/2)	0.896 (0.518–1.550)	0.694	1.175 (0.647–2.134)	0.597
EVL (high vs. low)	0.414 (0.24–0.713)	0.001	0.457 (0.256–0.818)	0.008

## Data Availability

The datasets presented in this study (sourced from TCGA and GEO) can be found in online repositories. The datasets sourced from clinical samples are available from the corresponding author on reasonable request.
